# The High-Affinity Phosphodiesterase BcPde2 Has Impact on Growth, Differentiation and Virulence of the Phytopathogenic Ascomycete *Botrytis cinerea*

**DOI:** 10.1371/journal.pone.0078525

**Published:** 2013-11-12

**Authors:** Karin Harren, Beate Brandhoff, Michael Knödler, Bettina Tudzynski

**Affiliations:** Westfälische Wilhelms-Universität Münster, Institute of Biology and Biotechnology of Plants, Münster, Germany; Seoul National University, Republic of Korea

## Abstract

Components of the cAMP signaling pathway, such as the adenylate cyclase Bac and the protein kinase A (PKA) were shown to affect growth, morphogenesis and differentiation as well as virulence of the phytopathogenic fungus *Botrytis cinerea*. While loss of Bac caused drastically reduced intracellular cAMP levels, deletion of the PKA resulted in extremely increased cAMP concentrations. To regulate the intracellular level of the second messenger cAMP, a balance between its biosynthesis through adenylate cyclase activity and its hydrolysis by phosphodiesterases (PDEs) is crucial. Here, we report the functional characterization of the two PDEs in the ascomycete *B. cinerea*, BcPde1 and BcPde2. While deletion of *bcpde2* resulted in severely affected vegetative growth, conidiation, germination and virulence, the *bcpde1* deletion strain displayed a wild-type-like phenotype. However, the double *bcpde1/2* deletion mutant exhibited an even stronger phenotype.

Localization studies revealed that BcPde2 accumulates at the plasma membrane, but is also localized in the cytoplasm. BcPde1 was shown to be distributed in the cytoplasm as well, but also accumulates in so far unknown mobile vesicles. Overexpression of *bcpde1* in the Δ*bcpde2* background rescued the deletion phenotype, and in addition an increased transcript level of *bcpde1* in the Δ*bcpde2* strain was observed, indicating redundant functions of both PDEs and an interdependent gene expression.

## Introduction

*Botrytis cinerea* is a phytopathogenic ascomycete causing gray mold disease in more than 200 plant species. Infection occurs through penetration followed by invasive growth, subsequent maceration of the plant tissue and generation of asexual conidia. In inappropriate conditions survival is ensured through formation of sclerotia, which can either germinate vegetatively or serve as female partner in sexual reproduction [1]–[3].

External signals, such as humidity, pH, osmotic stress or nutrient availability, have to be sensed and transduced via a diversity of signaling cascades, which can be activated via stimulation of a heterotrimeric G protein. Besides the Ca^2+^-signaling pathway [4]–[6], for example the Gα-subunit Bcg1 is able to stimulate the 3′-5′-cyclic adenosine monophosphate (cAMP)-mediated pathway. Activation of adenylate cyclase (AC) activity leads to formation of the second messenger cAMP, which is subsequently bound by the regulatory subunits of the protein kinase A (PKA). In its inactive state, the PKA is a heterotetramer formed by two catalytic and two regulatory subunits. Binding of cAMP induces the release of the catalytic subunits of the PKA which in turn may phosphorylate downstream targets such as transcription factors [7].

In *B. cinerea*, several components of this pathway, including two of the three Gα subunits (Bcg1 and Bcg3), the AC (Bac), and the catalytic and regulatory subunits of the PKA have been characterized in the last years. Bcg1 controls light-dependent conidiation, sclerotia formation and invasive growth in plant hosts [8], whereas Bcg3 was shown to regulate conidiation and carbon-source-induced germination [9]. The cytosolic cAMP concentration in both mutants was lower than that of the wild type [9], [10], indicating that both Gα-subunits stimulate Bac activity.

Like other fungi, *B. cinerea* possesses one gene encoding the regulatory PKA subunit (*bcpkaR*), and two genes encoding catalytic subunits (*bcpka1*, *bcpka2*). BcPka1 appears to be the major subunit whose deletion led to significant growth retardation, delayed germination and reduced *in vitro* sporulation, as it was also observed for the *bac* deletion mutant [11], [12]. However, in contrast to the Δ*bac* mutant which has lost the ability to form sclerotia and to sporulate *in planta*, Δ*bcpka1* mutants are able to conidiate during infection in a wild type-like manner and to form sclerotia. These significant differences between the Δ*bac* and Δ*bcpka1* mutants indicate that additional cAMP-binding proteins beside the PKA must exist which transduce signals to other downstream targets. Unexpectedly, deletion of BcPkaR did not result in a constitutively active PKA as in other fungi, but instead in reduced PKA activity and hyperaccumulation of cAMP [11].

cAMP is the key second messenger of this pathway and its synthesis and degradation needs tight regulation. Inactivation of cAMP to AMP is carried out by hydrolysis through phosphodiesterase (PDE) activity and counteracts and resets the cAMP/PKA cascade. *Saccharomyces cerevisiae* possesses two PDEs, Pde1 and Pde2, with unrelated primary sequences [13]. Pde1 is described as a low-affinity PDE that downregulates agonist-induced cAMP accumulation in a PKA-controlled negative feedback loop, whereas Pde2 (high-affinity) controls the basal intracellular cAMP level [14], [15]. Both PDEs are at least partially redundant [16]–[18]. In all studies performed so far, deletion of the high-affinity Pde2 revealed a much stronger phenotype than deletion of *pde1* [17], [18].

Not much is known so far about the role of PDEs in filamentous fungi. In *Neurospora crassa*, a *pde2* deletion mutant failed to produce any conidia indicating that cAMP turnover is required for the transition from aerial growth to proconidial chain formation [19]. Recently, two studies have been published on characterization of PDEs in the rice-blast fungus *Magnaporthe oryzae* [20], [21]. Deletion of both genes showed that PdeH (homolog of yeast Pde2) is a key regulator of asexual and pathogenic development, whereas PdeL (homolog of Pde1) had no obvious function. Loss of PdeH or of both PDEs resulted in elevated intracellular cAMP levels and reduced virulence [20]. Furthermore, both single deletion mutants exhibited defects in their conidial morphology and hyphal branching pattern [21].

In this work we characterized single and double knockout mutants of both phosphodiesterase-encoding genes, *bcpde1* and *bcpde2*, in *B. cinerea*. Our results show that BcPde2 has a strong impact on virulence, but also on vegetative growth and development. BcPde1 plays only a minor role, but is able to functionally complement the Δ*bcpde2* phenotype. In contrast to other fungi, deletion of BcPde2 resulted in reduced intracellular cAMP levels.

## Materials and Methods

### Strains and culture conditions

*B. cinerea* Pers.:Fr. B05.10 is an isolate from *Vitis* [22], [23], and was used as host strain for transformation. All *B. cinerea* strains used in this study are listed in Table 1. Wild-type and mutant strains were grown on several complex media: potato dextrose agar (Sigma-Aldrich Chemie, Steinheim, Germany) was supplemented with 10% homogenized leaves of French bean (*Phaseolus vulgaris*) (PDAB). Synthetic complete medium (CM) was prepared according to Pontecorvo *et al.* [24]. As minimal medium, GB5 (0.33% Gamborg B5 [Duchefa Biochemie BV, Haarlem, The Netherlands], 2% glucose) was used. For conidiation, strains were incubated for seven days at 20°C under light/dark (12 h/12 h) conditions; for sclerotia formation, three weeks in continuous darkness. Conidiogenesis was quantified by inoculation of CM agar plates with 0.5×10^5^ conidia. After three weeks, conidia were harvested, filtrated and the conidia production was determined by counting. Conidiation and sclerotia formation were documented using the SteREO Discovery V.20 microscope equipped with an AxioCam MRc camera (both Carl Zeiss MicroImaging GmbH, Jena, Germany).

**Table 1:** *Botrytis cinerea* strains used in this study.

*B. cinerea* strain	Characteristics	Reference
WT: B05.10	Isolate from *Vitis vinifera* (Germany); *MAT1-1*	[22], [23]
Δ*bcpde1*	B05.10, Δ*bcpde1*::*hph*, homokaryon	this study
Δ*bcpde2*	B05.10, Δ*bcpde2*::*hph*, homokaryon	this study
ΔΔ*bcpde1/2*	B05.10, Δ*bcpde1*::*hph*, Δ*bcpde2*::*nat*, homokaryon	this study
Δ*bcpde1*+*gfp*-*bcpde1*	B05.10, Δ*bcpde1*::*hph*, *gfp*-*bcpde1*::*nat*, heterokaryon	this study
Δ*bcpde2*+*gfp*-*bcpde2*	B05.10, Δ*bcpde2*::*hph*, *gfp*-*bcpde2*::*nat*, heterokaryon	this study
Δ*bcpde2*+*gfp*-*bcpde1*	B05.10, Δ*bcpde2*::*hph*, *gfp*-*bcpde1*::*nat*, heterokaryon	this study
Δ*bac*	B05.10, Δ*bac*::*hph*, homokaryon	[12]
Δ*bcpka1*	B05.10, Δ*bcpka1*::*hph*, homokaryon	[11]

For DNA and RNA preparations, mycelium was grown on CM agar with a cellulose acetate (cellophane) overlay.

### Germination assays

Germination assays were performed as described by Doehlemann *et al.* [9]. Time-course analyses of conidia germination was performed using an AxioObserver.Z1 microscope (Carl Zeiss MicroImaging GmbH, Jena, Germany). In this case, conidia were incubated in ibidi plates (μ-Dish^35 mm, high^ glass bottom, ibidi GmbH, Germany) and germination was monitored in bright field.

### Microscopic analyses

Fluorescence microscopy was carried out after incubation of conidia for 16–24 hours on glass slides in liquid GB5 medium supplemented with 0.132 g/l (NH_4_)_2_HPO_4_. Staining of cell wall structures was carried out by addition of 10 µl Calcofluor White (CFW) solution (0.1%, in 0.1 M Tris/HCl, pH 8.5).

Fluorescence and light microscopy was performed with an AxioImager.M2 microscope (Carl Zeiss MicroImaging GmbH, Jena, Germany). Differential interference contrast (DIC) microscopy was used for bright field images. GFP fluorescence was examined with filter set 38 (excitation BP 470/40, beam splitter FT 495, emission BP 525/50). Calcofluor-white staining was imaged using the filter set 49 DAPI shift free (excitation G 365, beam splitter FT 395, emission BP 445/50). Images were captured with an AxioCam MRm camera (Carl Zeiss MicroImaging GmbH, Jena, Germany). All microscopic images were analyzed using the Axiovision Rel 4.5 software package (Carl Zeiss MicroImaging GmbH, Jena, Germany).

### Virulence assays

For penetration assays on onion epidermal layers, epidermal strips were washed with double-distilled water and incubated for one hour in a humid chamber at 70°C. Conidia were harvested, washed three times with double-distilled water and diluted to a concentration of 5×10^4^ spores/ml. 10 µl-droplets of these suspensions were used for inoculation. After 24 h penetration was monitored microscopically with an AxioScope.A1 microscope (camera AxioCam MRc for color imaging, both Carl Zeiss MicroImaging GmbH, Jena, Germany). Extracellular fungal hyphae were stained using Lactophenol Aniline Blue solution (Sigma–Aldrich, Germany) prior to light microscopy.

Infection assays on primary leaves of *Phaseolus vulgaris* were performed with conidia from 7-day-old PDAB agar cultures as described previously [10]. Infected plants were incubated in a plastic propagator box at 20°C under natural illumination.

### Standard molecular methods

Fungal genomic DNA (gDNA) was isolated as described previously [25]. Southern blot analyses with fungal DNA were performed according to the method of Sambrook *et al.* [26]. Total RNA was isolated from mycelial samples using the Trizol procedure (Invitrogen, Groningen, Netherlands). 1 µg of total RNA was taken for cDNA synthesis using the oligo(dT)12–18 primer and SuperScript II reverse transcriptase (Invitrogen, Groningen, Netherlands) according to the manufacturer's instructions. To avoid gDNA amplification, RNA samples were treated with DNAseI (Promega). For sequence analyses, Lasergene v10 software (DNAStar, Madison, WI) was used. BLASTP analysis was performed using the website http://www.ncbi.nlm.nih.gov/blast/Blast.cgi.

All primers used in this study are listed in Table S1.

Quantitative real-time PCR was carried out using a one-tenth dilution of the cDNA template in a MyiQ2 Two-Color Real-Time PCR Detection system with the Bio-Rad iQ SYBR Green supermix (Bio-Rad, Hercules, CA, U.S.A). Genes encoding actin A (XP_001553368, primers 30&31), elongation factor 1-alpha (XP_001551786, primers 32&33) and tubulin (AAB60307, primers 34&35) showed the same expression pattern in wild-type and mutant strains used in this work and were therefore used to normalize the cDNA amounts in the samples. To study the expression of *bcpde1* and *bcpde2*, primer couples 15&16 and 28&29 were used. Annealing temperatures ranged from 58°C to 62°C, while extension times were fixed to 20 s. For each gene, the PCR efficiency was between 90 and 110%. The relative expression of *bcpde1* and *bcpde2* was calculated following the ΔΔCt (cycle threshold) Pfaffl method, from the mean of two different determinants of Ct values.

Statistical analyses were performed by independent comparison of the gene expression values in the mutant strains to those in the wild type in each condition by using two-sample t-tests in Excel (Microsoft).

### Generation of mutants

The replacement constructs for single deletion of *bcpde1* and *bcpde2* were generated using the homologous recombination system in yeast according to Colot *et al.* [27]. Therefore, about 1 kb of the 5′- and 3′-non-coding regions of both genes were amplified from gDNA of *B. cinerea* wild-type strain B05.10 using primer pairs 1&2 and 3&4 for *bcpde1* and 17&18 and 19&20 for deletion of *bcpde2* (Fig. S1A). The hygromycin resistance (*hph^R^*) cassette containing *hph* under control of the *trpC* promoter of *A. nidulans* was amplified with primers 5&6 using pCSN44 [28] as template. After linearization of pRS426 (restriction with EcoRI/XhoI), all fragments were co-transformed into uracil-auxotrophic *S. cerevisae* strain FY834 for assembly [27]. Total DNA from uracil-prototrophic yeast colonies was isolated with the GeneJET™ Plasmid Miniprep Kit (Fermentas). The replacement constructs were re-amplified from yeast DNA by PCR using the primers 1&4 (*bcpde1*) and 17&20 (*bcpde2*), respectively, and used for transformation of *B. cinerea* B05.10. Homologous integration events in hygromycin-resistant transformants were verified by diagnostic PCR using primers 7&8 for integration of the *hph^R^* cassette at the 5′ region, and 9&10 for the 3′ region (*bcpde1*) and 22&10 and 11&23 (*bcpde2*), respectively. Single-spore isolates were screened for absence of *bcpde1* and *bcpde2* alleles with the primer pairs 11&12 and 24&25, respectively. To show absence of further ectopic integrations, gDNA was used for Southern blot analysis. For deletion of *bcpde1*, gDNA was digested with BamHI, blotted, and hybridized with the 5′-flank of *bcpde1*. The replacement of the *bcpde1* allele by the *hph^R^* cassette leads to a hybridizing fragment of 3.8 kb, in comparison to the wild-type fragment of 5.8 kb (Fig. S1B). For single deletion of *bcpde2*, gDNA was restricted with BglII and the 3′-flank was used as probe. A hybridizing fragment of 4.6 kb was obtained for *bcpde2* deletion mutants while a hybridizing fragment of 6.7 kb was obtained for the recipient strain B05.10 (Fig. S1B).

For generation of mutants lacking both *bcpde1* and *bcpde2*, a second replacement fragment for *bcpde2* was cloned comprising the nourseothricin resistance (*nat^R^*) cassette from pZPnat1 according to the above mentioned strategy (Fig. S1A). The homokaryotic strain Δ*bcpde1*-T1 was transformed with the linearized replacement fragment (PvuI/PvuII). Transformants were selected on nourseothricin-containing medium. Diagnostic PCR to detect homologous integration (HI) was performed as described before using the primers 22&36 (5′ HI), 11&23 (3′ HI) and 24&25 (*bcpde2* allele). For Southern blot analysis, gDNA was restricted with EcoRI and hybridized with the 3′ flank (Fig. S1B). The replacement of *bcpde2* leads to a hybridizing fragment of >10 kb in the deletion mutants, in comparison to the wild type with 3.8 kb.

In each case, identical phenotypes for two or three independent transformants were observed. Hence, results are just presented for one mutant per construct.

For construction of *gfp*-*bcpde1* the coding region of *bcpde1* was amplified using the primers 13&14 that contain overlapping sequences homologous to the vector pNAN-OGG [29]. This vector contains flanks mediating the replacement of the gene encoding the nitrite reductase, and by this ensuring integration at a known locus, a *nat^R^* cassette, and a codon-optimized *gfp* [30] under control of the *oliC* promoter and the *gluc* terminator. The PCR product and the NotI-digested plasmid pNAN-OGG were co-transformed into *S. cerevisiae* yielding pNAN-OG*^bcpde1^*G. DNA of pooled yeast colonies was isolated as described above and transformed into *E. coli*. Plasmid DNA from single colonies was isolated and sequenced. pNDN-OG*^bcpde2^*G, to express *gfp*-*bcpde2*, was constructed similarly using primers 26&27 and pNDN-OGG as basic vector. For transformation, both vectors were linearized with SacII/ApaI. The resulting fragments contained the expression cassettes (P*oliC*, *gfp*-*bcpde1* or *gfp*-*pde2*, T*gluc*) and the *nat^R^* cassette surrounded by flanks mediating the homologous recombination at *bcniiA* or *bcniaD*. The *gfp*-*bcpde1* fusion construct was introduced in the wild type, Δ*bcpde1*-T1 and Δ*bcpde2*-T6, the *gfp-bcpde2* construct in the wild type and Δ*bcpde2*-T6. Integration of the whole fusion constructs and the resistance cassettes at *bcniiA* or *bcniaD* was performed by diagnostic PCR.

### Transformation of *B. cinerea*

Protoplast transformation was performed as described previously [31]. Resistant colonies were transferred to GB5-plates containing 70 µg/ml of hygromycin B (Invitrogen, San Diego, CA, USA) or 70 µg/ml of nourseothricin (Werner-Bioagents, Jena, Germany). Single conidial isolates were obtained by spreading conidial suspensions on selection plates. Germinated conidia were transferred individually onto new plates until homokaryotic mutants were obtained.

### PKA activity assay

Mycelia were grown for three days on CM- or GB5-agar plates with a cellophane overlay, harvested and ground under liquid nitrogen. Protein extraction was performed as described by Liebmann *et al.* [32]. Fluorescent dye-coupled kemptide peptide was used to determine PKA activity following the manufacturer's protocol (Pep-Tag Assay for Non-Radioactive Detection of cAMP-dependent Protein Kinase, Promega).

### cAMP assay

Cellular cAMP was measured using the highly sensitive Amersham cAMP Biotrak Enzymeimmunoassay (GE Healthcare Limited) following the manufacturer's instructions. Lyophilized mycelium was treated with lysis reagent 1B for 15 min and centrifuged for 10 min at maximum speed, and the supernatant was used in several dilutions (1∶5, 1∶10, 1∶50, and 1∶100) for the enzyme-linked immunosorbent assay.

## Results

### Identification of phosphodiesterases

By using the protein sequences of yeast Pde1 (CAA64139) and Pde2 (CAA99689) as queries for BlastP analyses, we identified two homologous proteins in the *B. cinerea* database (www.broadinstitute.org/annotation/genome/botrytis_cinerea): B0510_5711 and B0510_6935, respectively. The amino acid sequence of B0510_5711 revealed 35% identity (47% positives) to the yeast protein Pde1, whereas the B0510_6935 sequence revealed 26% identity (39% positives) to the yeast Pde2 protein. Exon-intron organizations of these genes (hereafter called *B. cinerea pde1* and *pde2*) were confirmed by sequencing of cDNA clones. Both PDEs contain conserved phosphodiesterase domains and share sequence similarities with PDEs of other fungi.

For functional analysis, single and double deletion mutants of both genes were generated (Fig. S1, Table 1). As independent transformants exhibited identical phenotypes the results for one arbitrarily chosen mutant per construct (Δ*bcpde1-*T1, Δ*bcpde2*-T6, ΔΔ*bcpde1/2*-T5) are shown.

### Deletion of *bcpde2* results in a drastic reduction of vegetative growth

**Figure 1:**
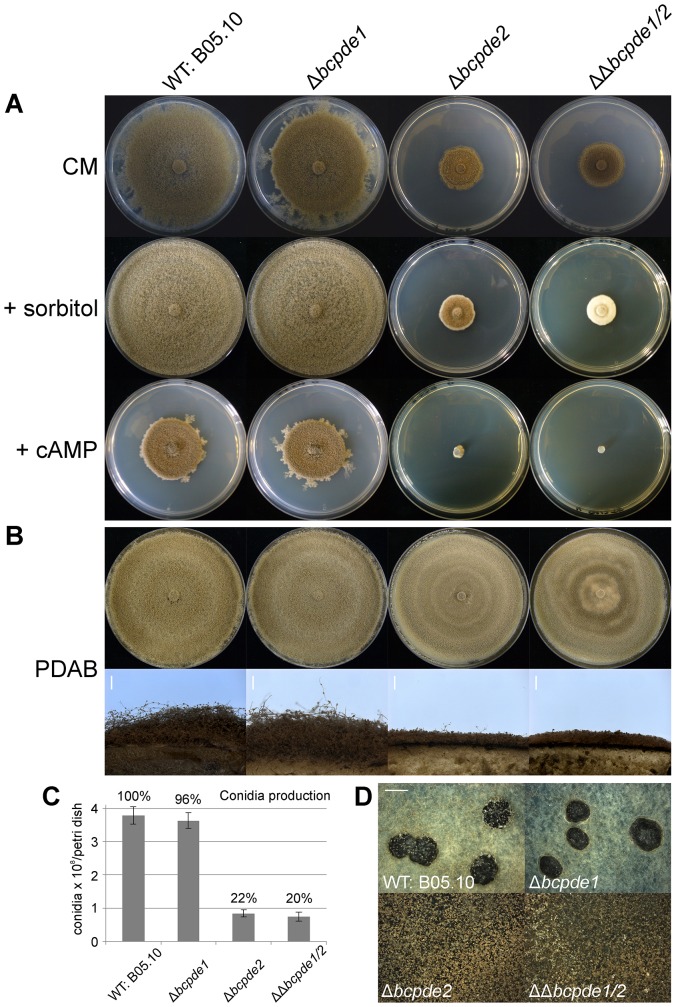
Colony morphologies and light-dependent development of the three *bcpde* knock-out mutants compared to the *B. cinerea* wild type. **A**: Colony morphology. Indicated strains were grown on complete medium (CM) supplemented with different compounds (1 M sorbitol or 3 mM cAMP) for seven days under light/dark conditions and 20°C. **B**: Light-dependent conidiation. Indicated strains were grown for seven days on PDAB agar plates. Upper panel: top view; lower panel: lateral view of cross sections. Scale bars = 1 mm. **C**: Quantification of conidia production. CM agar plates were inoculated with 5×10^4^ conidia and incubated for two weeks under light/dark conditions (12 h/12 h). Conidia were harvested and counted. Data represent the mean and standard deviations of six measurements (two petri dishes in three independent experiments per strain). Relative amounts of conidia are indicated in numbers compared to the WT (100%). **D**: Induction of sclerotial differentiation. Inoculated CM agar plates were incubated for three weeks in constant darkness. Scale bar: 2 mm.

To compare vegetative growth of the mutants and the wild type, all strains were grown on solid complete medium (CM) with or without different supplements (Fig. 1A). The *bcpde1* deletion strain displayed a wild-type-like growth pattern on all media, whereas mutants lacking *bcpde2* (Δ*bcpde2* and ΔΔ*bcpde1/2*) showed drastically reduced growth rates. After one week the respective strains displayed colony diameters of about three cm, whereas the wild type and *bcpde1* mutants exhibit growth rates of one cm per day. Osmotic stress generated by high sugar concentrations (1 M sorbitol) abolished conidia formation in Δ*bcpde2* and ΔΔ*bcpde1/2* mutants without affecting radial growth compared to basal CM. Addition of cAMP resulted in a decreased radial growth of all strains. The most striking effect was observed for the ΔΔ*bcpde1/2* mutant whose growth was completely blocked, whereas the single Δ*bcpde2* mutant was still able to grow though very slowly.

To investigate a possible involvement of the PDEs in conidiogenesis in more detail, we quantified the numbers of produced conidia on solid medium. While the Δ*bcpde1* mutant produced aerial mycelium and wild-type-like amounts of conidia, only about 20% of spores compared to the wild type were observed for the single and double *bcpde2* deletion mutants (Fig. 1B, 1C). However, in contrast to the wild type and Δ*bcpde1* strains, which formed dark-pigmented sclerotia when incubated for three weeks in continuous darkness, the Δ*bcpde2* and ΔΔ*bcpde1/2* mutants formed abundant conidia instead of sclerotia in the dark (Fig. 1D).

Summarizing, BcPde2 plays an essential role in vegetative growth, conidiation and sclerotia formation. As the double deletion mutant was under some conditions even more affected than the single mutants, an additive effect of both PDEs is suggested.

### BcPde2 has an impact on conidial shape, size and germination

**Figure 2:**
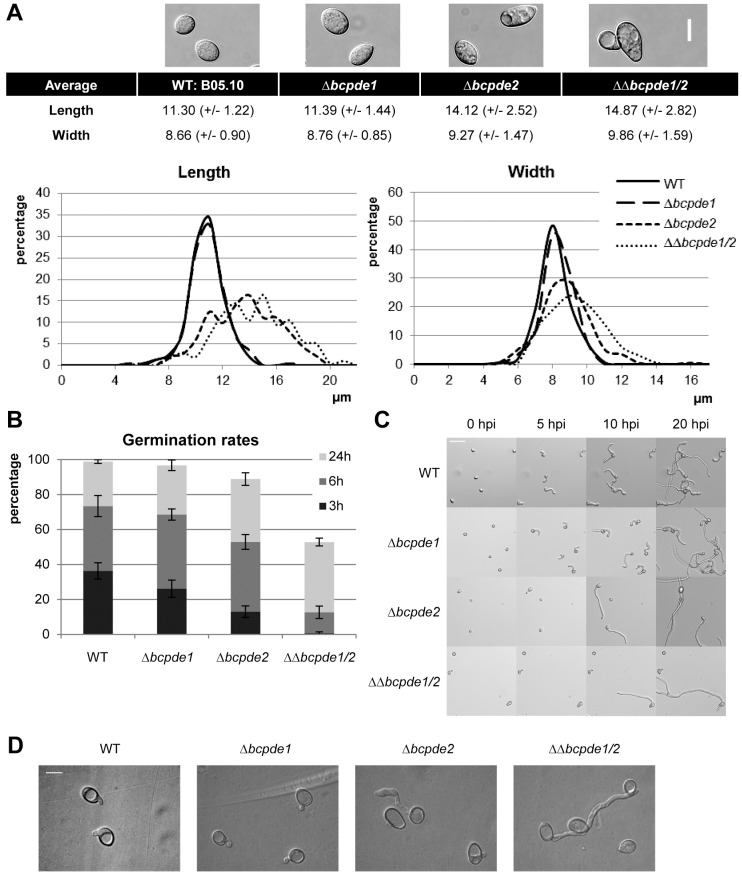
Conidia morphology and germination. **A**: Morphology and size of non-germinated conidia. Conidia suspensions (in water) were microscopically examined. Conidia diameters (length and width) were measured using the microscope software. Mean values and standard deviations were calculated with n spores (WT: n = 157, Δ*bcpde1*: n = 230, Δ*bcpde2*: n = 233, ΔΔ*bcpde1/2*: n = 201). Distributions are represented in relative amounts of spores (percentages) per µm. Scale bar = 10 µm. **B**: Germination rates of conidia suspensions in GB5+10.5 mM glucose on glass surface. Represented are mean values and standard deviations of triplicate counting in two independent experiments. **C**: Sugar-induced germination in GB5+10.5 mM glucose was monitored in a time-course experiment. Scale bar = 50 µm. **D**: Hydrophobicity-induced germination in double-distilled water was monitored on polypropylene foil. Images were taken after 24 h of incubation. Scale bar = 10 µm.

Microscopic studies showed that conidia of Δ*bcpde2* and ΔΔ*bcpde1/2* mutants differed in shape and size from those of the wild-type and seemed to be highly vacuolized (Fig. 2A). While wild-type conidia displayed an average length of about 11.3 µm and width of 8.7 µm, the *bcpde2* deletion resulted in a large variance of conidia size. In addition to the altered forms and diameters, Δ*bcpde2* conidia displayed delayed carbon source (10.5 mM glucose)-induced germination rates (Fig. 2B). However, after 24 hours, about 80% and 50% of the conidia from the single *bcpde2* and double *bcpde1/2* mutants, respectively, were able to germinate. Furthermore, the shape of the young germ tubes and hyphae was different in *bcpde2* mutants (Fig. 2C). Usually, the wild type conidia germinate after a stage of swelling within the first four to six hours and form an appressoria-like structure. In most cases, a second germ tube at the opposite side of the spore develops after a short break, and both hyphae grow in different directions and start branching. In contrast, to that, a majority of conidia of single and double *bcpde2* mutants exhibit just one germ tube which grew straightforward without formation of appressoria-like structures or further lateral curves.

Germination of *B. cinerea* conidia can also be induced by hydrophobic surfaces in the absence of nutrients in a MAPK-dependent manner [9]. The conidia of the Δ*bcpde1* mutant developed the characteristic wild-type-like short germ tubes on polypropylene. In contrast, the Δ*bcpde2* strain formed longer germ tubes, while the conidia of the double knock-out strain generated either long and thin germ tubes, or did not germinate at all (Fig. 2D).

In conclusion, BcPde2 has an impact on number and size of macroconidia and nutrient- and hydrophobicity-induced conidial germination. Since the ability to germinate and the mode of germination are important steps in the early stages of infection we studied the role BcPde1 and BcPde2 may play in virulence.

### BcPde2 is essential for full virulence

**Figure 3:**
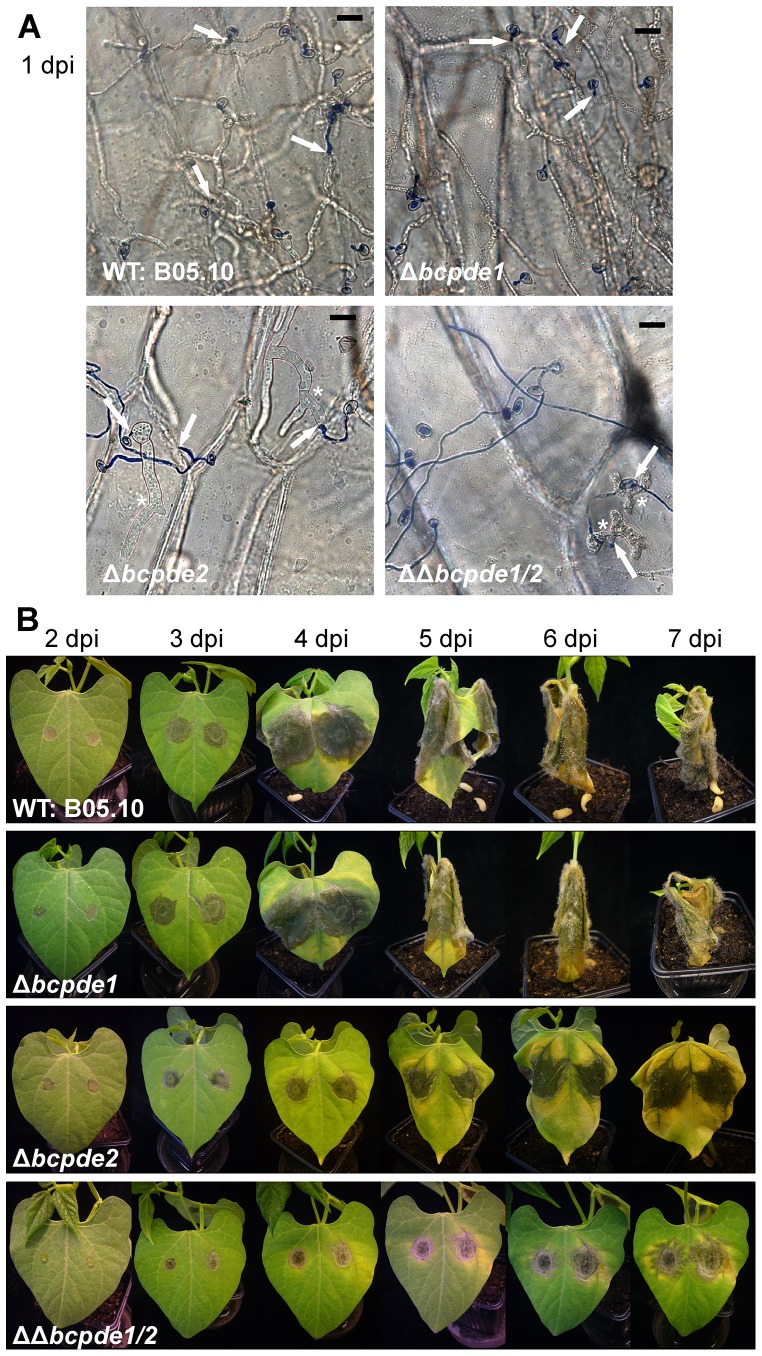
Virulence assay. **A**: Penetration assay on onion epidermis cells. The hydrophobic sides of onion epidermis cells were inoculated with conidia suspensions (in water) of the indicated strains. 22 h post inoculation top-layer hyphae were stained with lactophenol blue and analyzed microscopically. Intracellular hyphae were not stained. Conidia of the WT and Δ*bcpde1* strain formed short germ tubes that directly penetrated (arrows) the plant surface. The Δ*bcpde2* conidia germinated, and germlings penetrated (arrows), but within the plant cells, hyphae swelled (up to sixfold of the normal diameter) and further growing mycelium was thickened (asterisk). Some conidia of ΔΔ*bcpde1/2* did not germinate, some germinated, but after penetration (arrows), hyphae were strongly disturbed in development and swollen (asterisks). Scale bar: 20 µm. **B**: Infection of living beans and analyses of *in planta* development. Primary leaves were inoculated with droplets of conidial suspensions of the indicated strains. Images were taken after 2 to 7 days post infection (dpi).

First, we investigated the mutants' ability to penetrate onion epidermis cells (Fig. 3A). The Δ*bcpde1* strain developed one wild-type-like short germ tube (blue-stained) directly penetrating the onion cells. Some conidia derived from Δ*bcpde2* and ΔΔ*bcpde1/2* strains also formed germ tubes and penetrated the epidermis cells. However, many conidia of the double deletion mutant were either not able to germinate at all (similar to the observations in axenic culture), or they formed long germ tubes which grew on the surface without penetrating. In the rare cases, when hyphae penetrated the onion epidermis cells, a drastic effect was noticed: the hyphae of the Δ*bcpde2* single mutant thickened up to six fold of the normal hyphal diameter (Fig. 3A, asterisks). These hyphae were still able to branch and grew further in a wild type-like manner. The hyphae of the ΔΔ*bcpde1/2* strain also drastically thickened after penetration, but no further spreading was observed in any case.

When leaves of young bean plants were inoculated with conidia suspensions of the strains (Fig. 3B), both, the wild type and the Δ*bcpde1* mutant developed primary lesions (2 days post infection, dpi), followed by secondary spreading lesions (3 dpi) and full maceration of the plant tissue accompanied by formation of conidia (6 dpi). On the contrary, Δ*bcpde2* and ΔΔ*bcpde1/2* mutants were retarded in the infection process. Progression of infection stopped finally before reaching the soft rot stage. The ΔΔ*pde1/2* strain was even more delayed as primary lesions became only visible at 3 dpi. In accordance with the reduced virulence both Δ*bcpde2* and ΔΔ*bcpde1/2* mutants lost the ability to produce conidia during infection.

Summarizing these results, BcPde2 is essential for full virulence. Both, the onion epidermis and bean leave pathogenicity tests revealed no defect in host penetration, but a retardation of invasive growth within the plant tissue and loss of *in planta* conidiation.

### GFP-BcPde2 is localized in the cytoplasm and the plasma membrane

**Figure 4:**
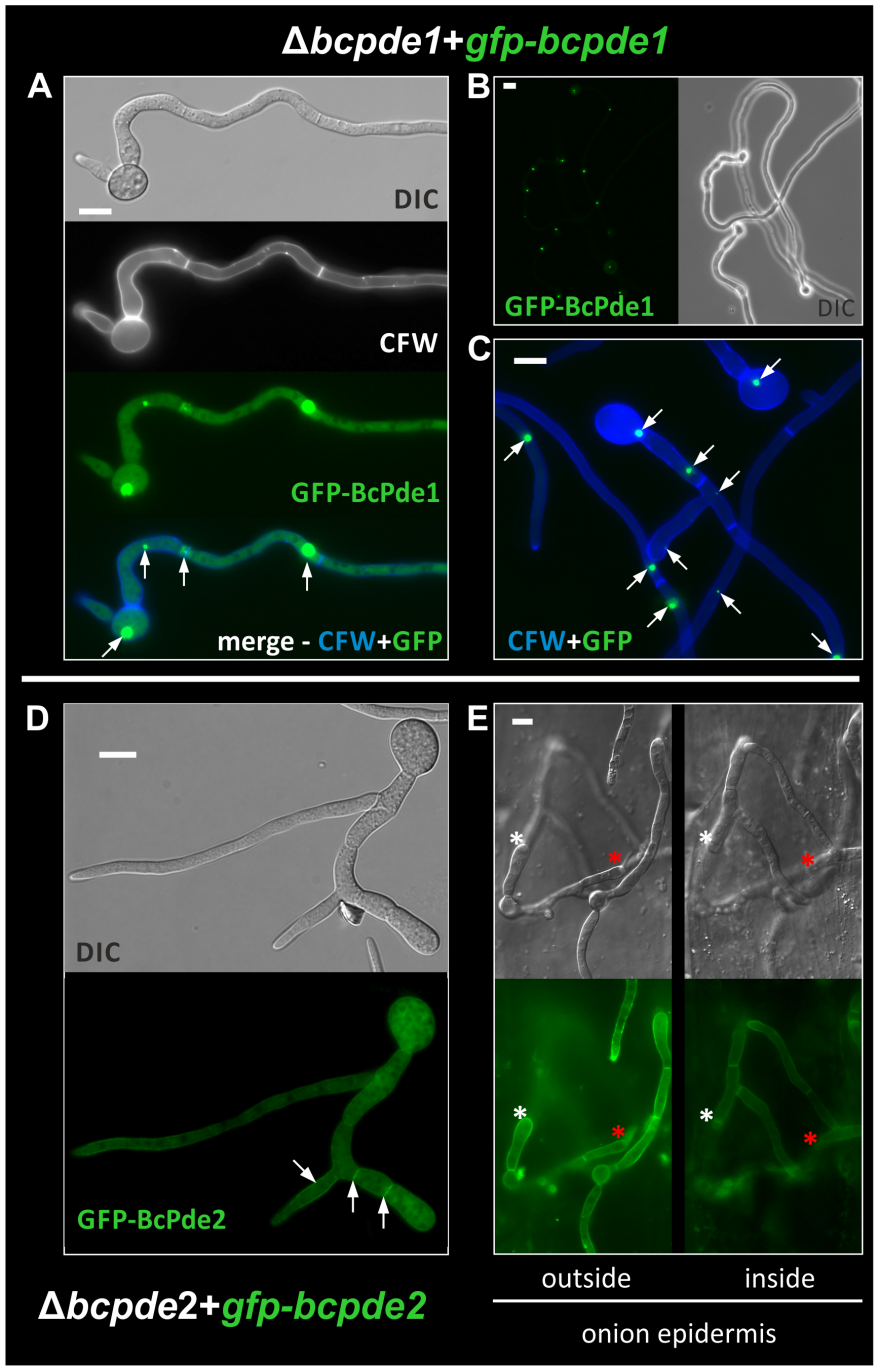
Localization studies of BcPde1 and BcPde2. **A**: Germinated conidia of strain Δ*bcpde1*+*gfp*-*bcpde1* were microscopically examined in sugar-inducing conditions on object slides. GFP-BcPde1 fluorescence was observed over the whole hyphae. A very strong fluorescent signal was detected in so far unknown mobile spots (white arrows), with different sizes. DIC: differential interference contrast image; CFW: Calcofluor white staining of fungal cell wall (white), merge: GFP and CFW overlay. **B**: GFP-BcPde1 fluorescence in germinated conidia (20× magnification). **C**: 40× magnification of *gfp*-*bcpde1* expressing germinated conidia. Overlay of CFW (blue) and GFP pictures. Low exposure time presents various sizes of unexplained spots (white) of GFP fluorescence in different cellular regions. **D**: Germinated conidia of strain Δ*bcpde2*+*gfp*-*bcpde2* were microscopically examined in sugar-inducing conditions on object slides. GFP-BcPde2 fluorescence was observed over the whole hyphae. A strong signal was detected at the plasma membrane and septa (white arrows). E: Conidia of strain Δ*bcpde2*+*gfp*-*bcpde2* were microscopically examined after penetration of onion epidermis cells. Left: top of the onion epidermis. Right: Hyphae growing inside the onion epidermis after penetration. Asterisks present points of penetration. Strong fluorescence was observed at the plasma membrane and the septa. The Δ*bcpde2* phenotype (swollen hyphae after penetration) was restored by expression of *gfp*-*bcpde2*. Scale bar = 10 µm.

In order to visualize the intracellular localization of the two PDEs, we generated GFP fusion constructs of both proteins under control of the constitutively expressed *oliC* promoter. The *gfp*-*bcpde1* fusion construct was expressed in two genomic backgrounds: wild type and Δ*bcpde1*. Both strains expressing the GFP-BcPde1 construct displayed a wild-type-like phenotype. Fluorescence was microscopically examined in germinating conidia on glass slides in the presence of glucose. Strong fluorescent signals were observed in small undefined vesicles in hyphae and conidia, while only weak signals were seen in the cytosol (Fig. 4A–C). Often just one strong, punctual fluorescent signal per compartment was visible, but sometimes also several smaller ones were present (Fig. 4B and 4C). Time-lapse analyses showed that the GFP-BcPde1 fusion protein was highly dynamic and probably associated to mobile vesicle-like bodies (data not shown).

Similarly, a *gfp*-*bcpde2* fusion construct was expressed in the wild type and the Δ*bcpde2* strain. All described phenotypes caused by deletion of *bcpde2*, e.g. the significantly reduced virulence on bean plants, were restored by expressing the fusion construct in the mutant background indicating its full functionality (Fig. S2). Expression of the *gfp*-*bcpde2* fusion construct in the wild-type background did not lead to phenotypic differences (data not shown). Localization of the fluorescent protein was examined in germinating conidia on glass slides as described above (Fig. 4D), and in water drops on onion epidermis strips (Fig. 4E). Invasively growing hyphae which have penetrated the onion epidermis cells showed wild-type-like growth. The GFP-BcPde2 fusion protein was evenly distributed throughout the cytosol in both conditions. However, fluorescence signals were enriched at the plasma membrane and septa.

Thus, BcPde1 and BcPde2 were found to be localized mainly in unknown, mobile vesicles, and to the plasma membrane and septa, respectively, suggesting a spatial distribution and function of both proteins.

### Loss of *bcpde2* activates *bcpde1* transcription

**Figure 5:**
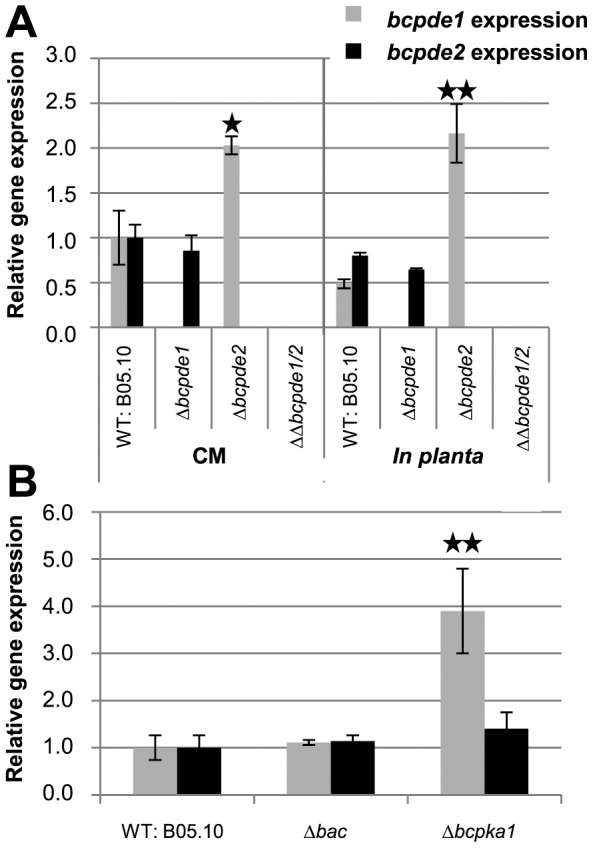
Interdependent gene expression analysis. The expression of *bcpde1* and *bcpde2* was measured by quantitative real time PCR. **A**: The *B. cinerea* wild type (WT) and the three different *bcpde* deletion strains were incubated for three days on solid complete medium (CM) with a cellophane overlay. For *in planta* material the same strains were used for conidia-derived infection of living bean plants. Whole leaves with many infections spots were harvested at the stage of primary infection (WT, Δ*bcpde1*: 50 hpi, Δ*bcpde2*, ΔΔ*bcpde1/2*: 72 hpi; approximately the same lesion stage). **B**: The WT and the *bac* and *bcpka1* deletion strains were incubated for three days on CM medium. As reference genes, the genes encoding actin (XP_001553368), beta tubulin (AAB60307) and elongation factor 1-alpha (XP_001551786) were used. The indicated values are means of three biological replicates, and were normalized to the expression in the wild type on CM. Standard deviations are indicated by error bars. Asterisks above the bars denote significant differences in the measurements compared to the WT in each condition. * = P<0.05; ** = P<0.01.

We examined the transcript levels of both PDE-encoding genes in the wild type and the deletion mutants by qRT-PCR under *in vitro* (CM agar with a cellophane overlay) and *in planta* conditions (Fig. 5A). For *in planta* expression studies infected bean leaves were harvested at equal infection stages (primary lesions). On CM agar, the transcript level of *bcpde1* was significantly increased about twofold in the *bcpde2* knock-out strain in both conditions, while *bcpde2* was similarly expressed in both, wild type and the Δ*bcpde1* mutant.

To investigate whether the expression of the PDE-encoding genes is affected in mutants with altered cAMP levels, we further measured the transcript levels in the Δ*bac* (reduced cAMP levels) and the Δ*bcpka1* (elevated cAMP level) mutants. RNA of both mutants and the wild type was extracted from mycelia grown on CM agar (Fig. 5B). Interestingly, the *bcpde1* expression was drastically increased in the *bcpka1* mutant, similarly to the situation in the Δ*bcpde2* mutant (Fig. 5A). In contrast, no alteration of *bcpde1* and *bcpde2* expression levels was observed in the Δ*bac* mutant.

### *Bcpde1*-expression rescues *bcpde2* deletion phenotype

As both proteins are intended to possess PDE activity, we expected them to have redundant functions. To show whether BcPde1 is able to take over some functions of BcPde2, we transformed the *gfp*-*bcpde1* construct (*bcpde1* is driven by the constitutive *oliC* promoter) into the Δ*bcpde2* mutant. The complemented strains (Δ*bcpde2*+*gfp*-*bcpde1*) displayed a wild-type-like phenotype regarding vegetative growth, virulence and sclerotia formation, indicating that the GFP-BcPde1 protein was functional and able to rescue the loss of BcPde2 (data not shown). Microscopic analyses revealed a similar fluorescence signal of GFP-BcPde1 (cytosol and mobile vesicle structures) as in the wild type (WT+*gfp*-*bcpde1*) or Δ*bcpde1* (Δ*bcpde1+gfp*-*bcpde1*) genomic backgrounds (data not shown).

### Loss of BcPde2 results in reduced intracellular cAMP levels

**Figure 6:**
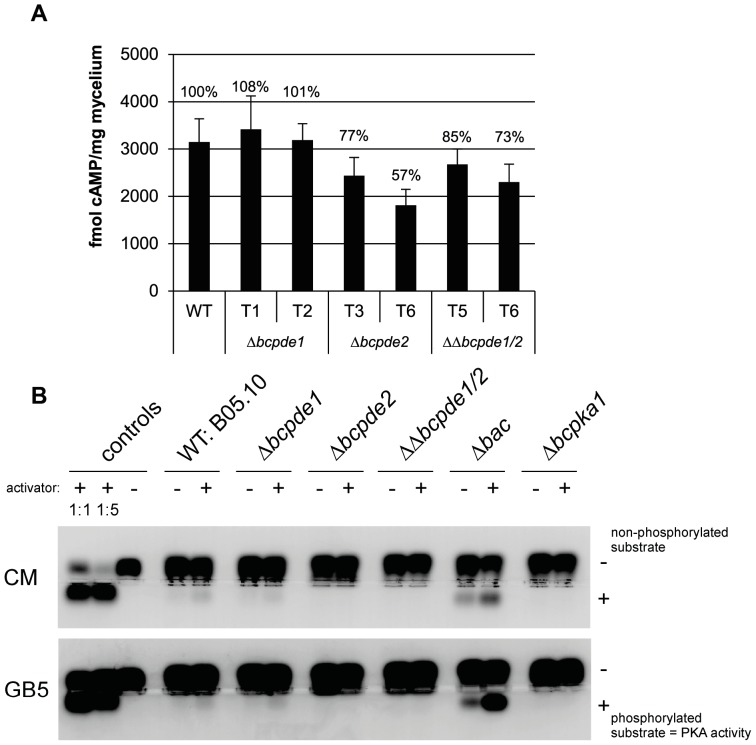
Quantification of cytosolic cAMP contents and PKA activity assay. **A**: Intracellular cAMP was determined in three day old mycelia cultures derived from GB5+cellophane cultures. Two independent primary transformants were tested for each deletion strain. Represented are mean values and standard deviations of technical duplicates in one experiment. Relative cAMP contents are indicated in numbers compared to the WT (100%). Three independent experiments revealed approximately analogue results. **B**: PKA activities from three day old mycelium cultures grown on CM+ or GB5+cellophane agar plates were monitored by gel electrophoresis according to Schumacher *et al.* [11]. The phosphorylated PKA substrate moved toward the anode (bottom). Samples with activator (+) contained cAMP.

As the phenotype of the *bcpde2* mutant deletion mutant might be due to altered cAMP levels, the cytosolic cAMP contents were quantified in vegetatively growing mycelium (Fig. 6A). Intracellular cAMP levels of the *bcpde1* deletion strains were similar to those of the wild type, while Δ*bcpde2* and ΔΔ*bcpde1/2* mutants showed slightly decreased cAMP levels. The Δ*bac* and Δ*bcpka1* strains served as controls exhibiting significantly decreased and increased cAMP contents, respectively (data not shown), as it was previously described [11]. To show, if the unexpected decrease of cAMP levels in the Δ*bcpde2* mutants affect the PKA activity, we performed a PKA activity assay. The test was performed under different conditions with mycelia grown on complete CM agar or synthetic GB5 agar. In both cases, no PKA activity was visible in the Δ*bcpde2* and ΔΔ*bcpde1/2* mutant strains, while the wild-type and the Δ*bcpde1* strains displayed similar PKA activities (Fig. 6B). Surprisingly, the Δ*bac* mutant showed significantly elevated PKA activity compared to the wild type independently of the growth conditions.

## Discussion

In *B. cinerea*, the Bac/cAMP/BcPka1-mediated signaling pathway was shown to play an important but not essential role in vegetative growth, differentiation and virulence [5], [8], [10]–[12]. However, there are still lots of unanswered questions regarding the regulation of cAMP levels in this fungus. Previously, we have shown that *B. cinerea* has some specific differences considering the cAMP signaling pathway compared to other fungi [11]. To further investigate how intracellular cAMP levels are regulated, we focused on the functional characterization of the two PDEs which are thought to degrade cAMP to AMP. Deletion of *bcpde2* caused severe phenotypes especially regarding vegetative growth, differentiation (formation of macroconidia and sclerotia), germination and virulence. Therefore, BcPde2, the homolog to the yeast high-affinity PDE, seems to be involved in regulation of many steps of the life-cycle in *B. cinerea*, while BcPde1 does not play an essential role in any of these processes. However, in some cases, the double knock-out strain showed an even more severe phenotype indicating an additive effect of both PDEs. Surprisingly, expression of the *gfp*-*bcpde1* construct could rescue all of the defects of the *bcpde2* deletion strain although the subcellular localization of both PDEs was different. Our suggestion that BcPde1 partially fulfills BcPde2-functions is supported by the observation that *bcpde1* expression was elevated in the Δ*bcpde2* mutant strain. Taken together, we suggest that both proteins are functionally connected due to their additive effect and interdependent regulation.

### BcPde2 controls differentiation in *B. cinerea*

Similarly to *M. oryzae* [20], BcPde1 is dispensable for vegetative growth, while BcPde2 is essential for colony extension and regulation of differentiation processes, such as conidiogenesis in light and sclerotia formation in darkness. Deletion of *bcpde2* resulted in light-independent conidiation and total loss of sclerotia formation in constant darkness indicating that BcPde2 plays an important role in light response and light-dependent differentiation processes. In contrast, the *N. crassa pde2* deletion mutant was completely blocked in conidial morphogenesis [19], while loss of PdeH in *M. oryzae* led to enhanced conidiation [20]. However, double deletion of both PDEs in *M. oryzae* resulted in total block of conidiation similarly to the *B. cinerea* ΔΔ*bcpde1/2* strain. As the conidia of the Δ*bcpde2* and ΔΔ*bcpde1/2* strains also varied in shape and size from that of the wild type, we conclude that BcPde2 is probably involved in cell wall and membrane stabilization as it was shown for Pde2 in *Candida albicans* [33]. However, in contrast to *C. albicans*, no differences were observed in shape or thickness of the cell wall and septa of the *B. cinerea* mutants stained with Calcofluor white (data not shown).

In *B. cinerea*, germination can be induced either by the presence of nutrients (on hydrophilic surfaces) or by hydrophobic surfaces (in the absence of nutrients), respectively [9]. Under the first condition, conidia develop thick and fast growing germ tubes, while small nose-like germ tubes are formed on hydrophobic surfaces. While nutrient-induced germination is mainly mediated via the cAMP signaling pathway through activation of Bcg3 and Bac, hydrophobicity-induced germination is regulated by the MAP kinase Bmp1 in a cAMP-independent manner [9], [11]. Here, we clearly demonstrate that BcPde2 is involved in both, the regulation of nutrient- and hydrophobicity-induced germination. The fact that hydrophobicity-induced germination is heavily affected, suggests that BcPde2 is probably involved in surface sensing, similarly to PdeH in *M. oryzae* which regulates surface sensing and guides germ tube growth during the early infection stages [20].

### BcPde2 plays an important role during infection

The data presented in this work clearly show that BcPde2 is required for full virulence. While the Δ*bcpde2* mutant seems to penetrate in a wild-type-like manner, invasive growth is affected in onion epidermis cells as well as on living bean plants. Delayed lesion formation and retardation of the whole infection process has already been demonstrated for other *B. cinerea* strains with mutations in the cAMP pathway, such as Δ*bac*, Δ*bcpkaR* and Δ*bcpka1* [11], [12].

Furthermore, in the *bcpde2* deletion strains, conidiogenesis was not only abolished under *in vitro* conditions but also completely inhibited during infection as it was also shown for the Δ*bac* mutant probably due to the reduced cAMP level in both mutants [11], [12]. In *M. oryzae* PdeH was shown to be needed during two critical steps of pathogenesis: infection-structure (appressoria) formation and invasive growth. However, in contrast to *B. cinerea bcpde2* deletion mutants, deletion of *pdeH* resulted in elevated and not reduced cAMP levels [20].

*In planta* expression studies of both PDE-encoding genes in the wild type revealed expression of both genes already in early infection states (6 to 24 hpi) as well as during primary lesion formation (data not shown).

### BcPde2 is responsible for maintenance of normal cAMP levels

Our speculation was that all the altered characteristics of the *bcpde2* mutants are due to an abnormal intracellular cAMP level as the mutants displayed an intermediate phenotype between the Δ*bac* (low cAMP levels) and the Δ*bcpka1* (high cAMP levels) mutant. Unexpectedly, the cAMP content was not increased in any *bcpde* deletion strain in contrast to other fungi. We observed wild-type-like concentrations for the Δ*bcpde1* strain, but reduced levels for Δ*bcpde2* and ΔΔ*bcpde1/2* mutants in three independent cAMP measurement approaches. This might be caused by an agonist-induced feedback loop in the cAMP signaling cascade due to a deregulated PKA activity in these mutants. A PKA activity assay with *B. cinerea* strains revealed no PKA activity at all in the Δ*bcpde2* and ΔΔ*bcpde1/2* strains. Thus, deletion of *bcpde2* resulted in a decrease of the intracellular cAMP level and in accordance to this, absence of PKA activity. These contrary effects on cAMP level compared to the *pdeH* mutant in *M. oryzae* cannot be explained so far.

Quantification of the *bcpka1* transcript level by qRT-PCR (data not shown) revealed no differences between the Δ*bcpde1*, Δ*bcpde2*, ΔΔ*bcpde1/2* and Δ*bac* strains compared to the wild type indicating that the reduced PKA activity in *bcpde2* mutants cannot be explained by reduced transcript levels. An interesting result was the significantly increased PKA activity in the Δ*bac* mutant which was in contrary to our expectation for a mutant with very low cAMP level (Fig. 6B).

In *S. cerevisiae* and *C. neoformans*, Pde1 is a target of PKA phosphorylation and is therefore involved in PKA-mediated feedback-regulation of cAMP level [34]. Sequence analyses distinguished a putative PKA-phosphorylation site at a threonine residue (KRGT^363^) in the BcPde1 protein sequence according to predicted phosphorylation sites for yeast PKA substrates [35]. However, a split-ubiquitin based yeast-two hybrid assay with BcPka1 and BcPde1/BcPde2 did not reveal any indication for an interaction between BcPka1 and BcPde1 or BcPka1 and BcPde2 (data not shown). Yet, we cannot exclude that maybe the second subunit BcPka2 is crucial for the proposed feedback regulation. Furthermore, Hicks *et al.* [34] proposed that in addition to the feedback regulation via cAMP degradation another system involves the cAMP production through repression of the AC. This potential dual regulation for maintenance of transient cAMP spikes is still not well understood in fungi, in general. Therefore, future studies may focus on investigations on the temporal regulation of the cytosolic cAMP concentration in *B. cinerea*.

### Localization studies revealed putative compartmentalization of cAMP response

Localization studies on the two PDE proteins revealed first hints for a compartmentalized cAMP distribution in *B. cinerea*, as shown for mammals [36]. This allows spatially distinct pools of PKA to be differentially activated. The basis for this is that PKA isoforms are anchored at specific intracellular sites by A-kinase anchoring proteins (AKAPs) [37], [38]. Therefore, discrete PKA populations could respond to gradients of cAMP and by this modify the activities of localized target proteins. The source for these gradients depends largely on PDE activities which can control intracellular diffusion of cAMP by their localization to specific subcellular compartments. PDE localization occurs through different mechanisms involving direct binding to membrane lipids or protein-protein interactions [36], [39].

We expressed *gfp* fusion constructs of both *B. cinerea* PDEs to gain information about their intracellular localization and proposed function. BcPde2 was clearly shown to be distributed in the cytosol, but also accumulates in the plasma membrane. The GFP-BcPde2 fusion protein fully restored wild-type-like growth under different conditions, the ability to form sclerotia in darkness, and full virulence. BcPde1 was also found to be localized in the cytoplasm, but strong fluorescent signals appeared in several mobile spots indicating an association of BcPde1 to mobile vesicle-like bodies. These observations suggest a differential regulation and function of both proteins and by this, a compartmentalization of distinct cAMP pools throughout the whole hyphae.

Localization studies of both PDEs (PdeH and PdeL) in *M. oryzae* showed cytosolic distribution of PdeH with a dynamical association to the plasma membrane and vesicular compartments, whereas PdeL seems to be localized predominantly in the nucleus [20]. These data demonstrate a differential compartmentalization of both PDEs. Compartmentalized cAMP signaling may be important for polarized growth of germ tubes, appressoria and infection hyphae of *M. oryzae*. In this kind of cells, relevant changes in cAMP levels could be controlled in space and time and anchored within subcellular compartments [20].

We assumed that maybe nutritional signals control the localization of both PDEs. However, addition of several stressors (e.g., cAMP or glucose) did not influence the PDE localization in *B. cinerea* (data not shown).

Despite the minor impact of BcPde1 on growth and differentiation, this protein seems to have similar functions as BcPde2. If the major BcPde2 is lost, the transcriptional level of *bcpde1* is elevated to compensate for the missing BcPde2 activity (Fig. 5). Similar observations were made in *M. oryzae* [20]. In addition, insertion of the *gfp*-*bcpde1* construct under control of a highly expressed promoter into the Δ*bcpde2* strain resulted in restoration of wild-type-like phenotype indicating that both PDEs have at least partly similar intracellular functions.

Interestingly, the *bcpde1* transcript level was also strongly induced in the Δ*bcpka1* background (Fig. 5B), probably caused by feedback regulation due to the strongly enhanced intracellular cAMP levels obtained in this mutant. As a low-affinity PDE, BcPde1 is proposed to down-regulate these high levels of the second messenger. The loss of BcPka1 resulted in enhanced cAMP and *bcpde1* transcript levels. However, the surplus of cAMP cannot be degraded probably due to the missing functional activation of the PDEs through PKA phosphorylation.

In summary, functional characterization of the two PDEs in *B. cinerea* provides another example for the differences in cAMP signaling between *B. cinerea* and other fungi such as *N. crassa* and *M. oryzae*. More studies have to be done to explain the unexpected down-regulation of cAMP levels in *bcpde2* mutants in contrast to the situation in *M. oryzae*, the unexpected loss of PKA activity in these mutants, and the increased PKA activity in the Δ*bac* mutant.

### Conclusions

**Figure 7:**
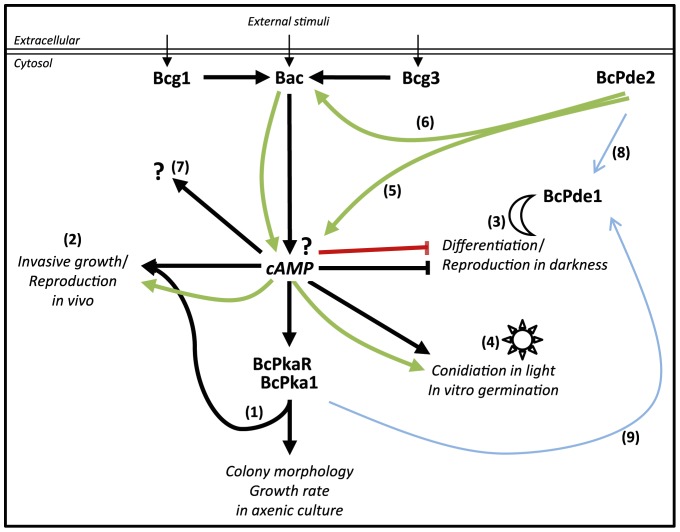
Schematic model of the postulated cAMP signaling network in *B. cinerea*. Activation of the adenylate cyclase Bac through the Gα subunits Bcg1 and Bcg3 leads to cAMP synthesis, which is suggested to further transduce incoming signals to the protein kinase A, composed of the regulatory subunit BcPkaR and the major catalytic subunit BcPka1. Different mutations in the mentioned signaling components were shown to have impact on the listed differentiation processes [11], [12]. Characterization of the phosphodiesterase BcPde2 led to this model, demonstrating positive or negative effects of this protein on cAMP signaling. An interconnection with BcPde1 on signal transduction and transcriptional regulation was further shown. Numbers in brackets are further described in the text.

In this study, we demonstrated that the high-affinity phosphodiesterase BcPde2 has great impact on growth, differentiation and virulence, whereas BcPde1 (low-affinity) plays only a minor role in these processes. Based on the presented data, we developed a model of the cAMP-mediated signaling pathway in *B. cinerea* and improved older models by integrating both PDEs (Fig. 7, (**1**)–(**8**)). According to this model, Bac is activated by the Gα subunits Bcg1 and Bcg3 resulting in increased cAMP levels and subsequent elevation of PKA activity (**1**). Several mutants of the cAMP pathway (Δ*bac*, Δ*bcg1*, Δ*bcg3*, Δ*bcpde2*) have some features in common: a delay of invasive growth and reproduction during infection (**2**), a light-independent conidiation and loss of sclerotia formation in the dark (**3**). Furthermore, the light-dependent conidiation, nutrient-dependent germination and surface sensing processes are controlled through Gα (Bcg3) and Bac activity (**4**).

Unexpectedly, the cAMP level was decreased in the *bcpde2* deletion mutants (**5**), and in accordance, PKA activity was completely absent, while it is increased in the Δ*bac* mutant. So far, we have no explanation for this and propose an unknown functional link between Bac, BcPde2 and BcPka1 (**6**). Furthermore, differences between Δ*bac* and Δ*bcpka1* mutants regarding *in planta* conidiation indicated that cAMP must be partially bound by other yet unknown cAMP binding proteins (**7**) beside BcPkaR. Interestingly, inactivation of BcPde2 causes a transcriptional up-regulation of *bcpde1* (**8**), and BcPde1 is able to restore wild-type-like growth and differentiation of the *bcpde2* mutant, when the *bcpde1* gene is overexpressed in the Δ*bcpde2* background indicating that both PDEs are functionally related. Furthermore, *bcpka1* deletion caused an increasing *bcpde1* transcript level (**9**).

*Botrytis*-specific differences in the cAMP pathway have already been observed before. As mentioned above, Δ*bcpka1* and Δ*bcpkaR* strains displayed almost identical phenotypes regarding the elevated cAMP levels, and *bcpkaR* deletion did not result in a strain with a constitutively active PKA [11], [12]. Furthermore, the Δ*bcpka1* mutant differs from Δ*bac* regarding light-regulated conidiation and *in planta* conidiation suggesting that additional cAMP binding proteins must exist. Recently, we deleted two genes encoding proteins with highly conserved cAMP-binding domains which could act in a BcPka1-independent cAMP-dependent signaling pathway (unpublished data). The functional analysis of these two mutant strains is in progress. Furthermore, nothing is known so far, about an influence of compartmentalization of the cAMP signal in filamentous fungi. Of course, distinct spikes of the second messenger can lead to various effects dependent on the intracellular localization.

## Supporting Information

Figure S1 **Gene replacement strategies and Southern blot analyses of** ***B. cinerea*** **strains Δ** ***bcpde1*** **, Δ** ***bcpde2*** **and ΔΔ** ***bcpde1/2*** **.** **A**: Strategies for generation of Δ*bcpde1* (top), Δ*bcpde2* (bottom) and ΔΔ*bcpde1/2* (middle) strains. All primers used for cloning of the replacement vectors and the diagnostic PCR analyses for proving homologous integration are indicated with numbers (1–25) and further described in the materials and methods section. Introns are depicted as white bars in arrows illustrating the genes. Restriction sites for Southern blot analyses are depicted. Top: Physical maps of *bcpde1* wild type (WT: B05.10) and Δ*bcpde1* locus. The wild type *B. cinerea* B05.10 was transformed with the *bcpde1* knock-out fragment (consisting of both flanking regions and the *hph^R^* resistance cassette derived from vector pCSN44) resulting in Δ*bcpde1* mutants via homologous recombination and insertion of *hph^R^*. Bottom: Physical maps of *bcpde2* in WT: B05.10 and replacement of *bcpde2* by *hph^R^* resistance cassette yielding Δ*bcpde2*. Middle: Replacement of *bcpde2* by the *nat^R^* resistance cassette in strain Δ*bcpde1* resulted in ΔΔ*bcpde1/2* mutants. **B**: Southern blot analyses of Δ*bcpde1*, Δ*bcpde2* and ΔΔ*bcpde1/2* mutants. Three independent mutants were tested for additional ectopic integrations of the replacement fragments. The wild type (WT), Δ*bcpde1* T1, T2, T3, all Δ*bcpde2* mutants T1, T6, T19 and ΔΔ*bcpde1/2* strains T5 and T6 displayed each just one hybridizing fragment with the expected size after hybridization with the probe (see 1A and materials and methods section).
(TIF)

Figure S2 **Infection of living beans and analyses of** ***in planta*** **development.** Primary leaves were inoculated with droplets of conidial suspensions of the indicated strains. The *gfp* fusion constructs were able to restore the wild-type phenotype of the Δ*bcpde2* mutant strain. Images were taken after 2 to 7 days post infection (dpi).
(TIF)

Table S1 **All primers used in this study.**
(DOCX)
