# Supplementary figures and images for: The High-Affinity Phosphodiesterase BcPde2 Has Impact on Growth, Differentiation and Virulence of the Phytopathogenic Ascomycete Botrytis cinerea
Source: PLoS One. 2013 Nov 12;8(11):e78525. doi: 10.1371/journal.pone.0078525 (PMC3827054; doi:10.1371/journal.pone.0078525)

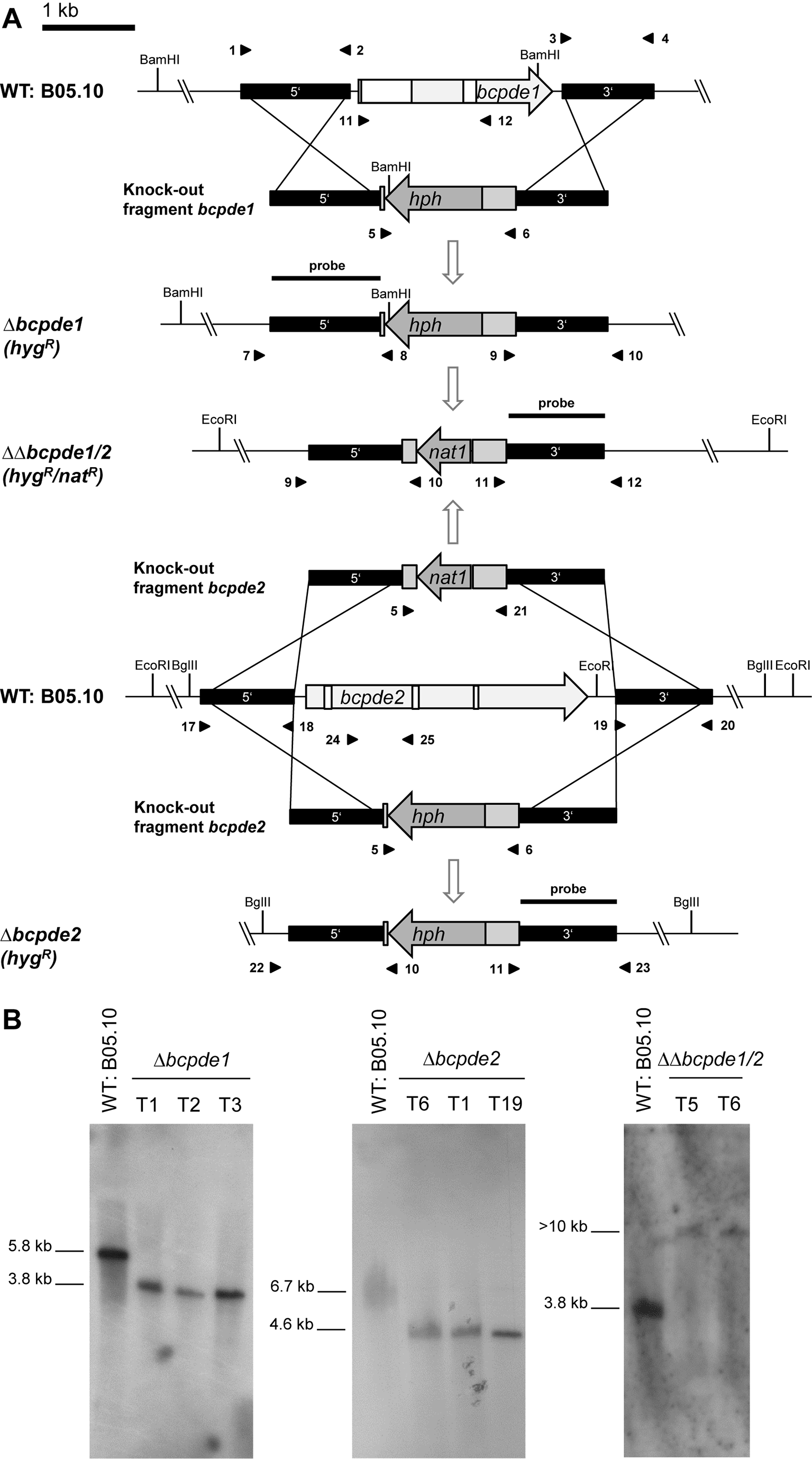

Supplement: Figure S1 — Gene replacement strategies and Southern blot analyses of B. cinerea strains Δ bcpde1 , Δ bcpde2 and ΔΔ bcpde1/2 . A: Strategies for generation of Δbcpde1 (top), Δbcpde2 (bottom) and ΔΔbcpde1/2 (middle) strains. All primers used for cloning of the replacement vectors and the diagnostic PCR analyses for proving homologous integration are indicated with numbers (1–25) and further described in the materials and methods section. Introns are depicted as white bars in arrows illustrating the genes. Restriction sites for Southern blot analyses are depicted. Top: Physical maps of bcpde1 wild type (WT: B05.10) and Δbcpde1 locus. The wild type B. cinerea B05.10 was transformed with the bcpde1 knock-out fragment (consisting of both flanking regions and the hphR resistance cassette derived from vector pCSN44) resulting in Δbcpde1 mutants via homologous recombination and insertion of hphR. Bottom: Physical maps of bcpde2 in WT: B05.10 and replacement of bcpde2 by hphR resistance cassette yielding Δbcpde2. Middle: Replacement of bcpde2 by the natR resistance cassette in strain Δbcpde1 resulted in ΔΔbcpde1/2 mutants. B: Southern blot analyses of Δbcpde1, Δbcpde2 and ΔΔbcpde1/2 mutants. Three independent mutants were tested for additional ectopic integrations of the replacement fragments. The wild type (WT), Δbcpde1 T1, T2, T3, all Δbcpde2 mutants T1, T6, T19 and ΔΔbcpde1/2 strains T5 and T6 displayed each just one hybridizing fragment with the expected size after hybridization with the probe (see 1A and materials and methods section). (TIF) [file pone.0078525.s001.tif]

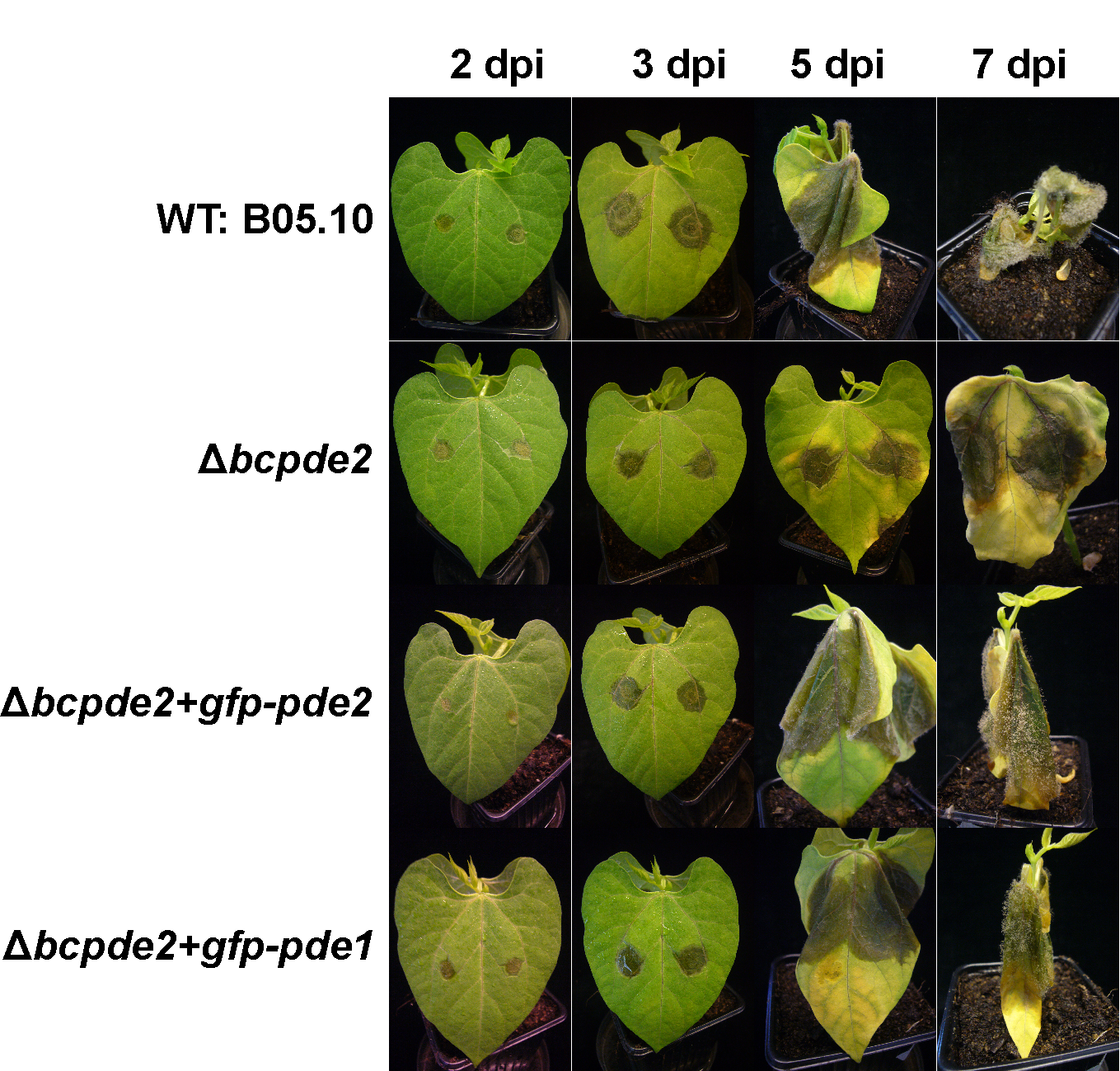

Supplement: Figure S2 — Infection of living beans and analyses of in planta development. Primary leaves were inoculated with droplets of conidial suspensions of the indicated strains. The gfp fusion constructs were able to restore the wild-type phenotype of the Δbcpde2 mutant strain. Images were taken after 2 to 7 days post infection (dpi). (TIF) [file pone.0078525.s002.tif]
