# Supplementary material for: The High-Affinity Phosphodiesterase BcPde2 Has Impact on Growth, Differentiation and Virulence of the Phytopathogenic Ascomycete Botrytis cinerea
Source: PLoS One. 2013 Nov 12;8(11):e78525. doi: 10.1371/journal.pone.0078525 (PMC3827054; doi:10.1371/journal.pone.0078525)
Supplement: Table S1 — All primers used in this study. (DOCX) [file pone.0078525.s003.docx]

**TABLE S1** All primers used in this study

| Gene | Purpose | No. | Name | Nucleotidesequence |
| --- | --- | --- | --- | --- |
| *bcpde1* | Deletion of *bcpde1* | 1) | PDE1_5F_YKO | GTAACGCCAGGGTTTTCCCAGTCACGACGTGGATGACGATGATGACTGG |
|  |  | 2) | PDE1_5R_YKO | ATCCACTTAACGTTACTGAAATCTCCAACACGTATCAAGACGTTGCTCG |
|  |  | 3) | PDE1_3F_YKO | CTCCTTCAATATCATCTTCTGTCTCCGACTGCCTGGAGGAAGGTAATAC |
|  |  | 4) | PDE1_3R_YKO | GCGGATAACAATTTCACACAGGAAACAGCATCGATCCCTACGAAGAAGG |
|  |  | 5) | hphF-trpC-P (hphF) | GTCGGAGACAGAAGATGATATTGAAGGAG |
|  |  | 6) | hphR-trpC-T (hphR) | GTTGGAGATTTCAGTAACGTTAAGTGGAT |
|  | HI *bcpde1* 5' | 7) | PDE1_5F_HI | TGATCAGTGGAGTTTGCCGAGTCAG |
|  |  | 8) | pCSN44-trpC-T | GGAATAGAGTAGATGCCGACCGG |
|  | HI *bcpde1* 3' | 9) | pCSN44-trpC-P | CCTCCACTAGCTCCAGCCAAGCCC |
|  |  | 10) | PDE1_3R_HI | TCCCTCCTGGAAACTCGGATCTG |
|  | *bcpde1* allele | 11) | PDE1_F_WT | AGCTCCCGCTGGGAATTCTATG |
|  |  | 12) | PDE1_R_WT | TTCGTTGGAGTGATTGGTTCCTAG |
|  | *gfp* fusion | 13) | bcpde1-gfp-R | CATACATCTTATCTACATACGATGGAAGGTAGAGCTCCCGC |
|  |  | 14) | bcpde1-gfp-F | GGGAATGGATGAACTTTACAAACTACAAATAAACACTCATCC |
|  | qRT-PCR | 15) | pde1_rt_pcr_F2 | GGTTATATGGAGATTGTAGAAGGACTGGCCG |
|  |  | 16) | pde1_rt_pcr_R2 | CGTTGGAGTGATTGGTTCCTAGTTGATAAGG |
| *bcpde2* | Deletion of *bcpde2* | 17) | PDE2_5F_YKO | GTAACGCCAGGGTTTTCCCAGTCACGACGTCTACACTTCCATTGCGACG |
|  |  | 18) | PDE2_5R_YKO | ATCCACTTAACGTTACTGAAATCTCCAACATTGATTGAGCTCGACAGCC |
|  |  | 19) | PDE2_3F_YKO | CTCCTTCAATATCATCTTCTGTCTCCGACACTTCTTCTTCTGGCATGGG |
|  |  | 20) | PDE2_3R_YKO | GCGGATAACAATTTCACACAGGAAACAGCTAGCATTTCATGGTGAGCCG |
|  |  | 5) | hphF-trpC-P (hphF) | GTCGGAGACAGAAGATGATATTGAAGGAG |
|  |  | 6) | hphR-trpC-T (hphR) | GTTGGAGATTTCAGTAACGTTAAGTGGAT |
|  |  | 21) | hphR-trpC-T2 | GTTGGAGATTTCAGTAACGTTAAGTGGATCGTATCTTATCGAGATCCTGAACACC |
|  | HI *bcpde2* 5' | 22) | PDE2_5F_HI | ACTCGACCTCGACTTAAACCCAAG |
|  |  | 10) | pCSN44-trpC-T | GGAATAGAGTAGATGCCGACCGG |
|  | HI *bcpde2* 3' | 11) | pCSN44-trpC-P | CCTCCACTAGCTCCAGCCAAGCCC |
|  |  | 23) | PDE2_3R_HI | TATGACGGTCACCAATGTAACCG |
|  | *bcpde2* allele | 24) | PDE2_F_WT | TGCAACATTGTCTACGTGGATCGC |
|  |  | 25) | PDE2_R_WT | TATTCTGGAAGGCTACCTAAGCGG |
|  | *gfp* fusion | 26) | bcpde2-gfp-F | GGGAATGGATGAACTTTACAAAATGGACTACGCCGCATGCAACATTG |
|  |  | 27) | bcpde2-gfp-R | CATACATCTTATCTACATACGTCAACCCGCAGTCCCATCAGAC |
|  | qRT-PCR | 28) | pde2-rt-pcr-F | GTGCAGACATAAGTAACGTGGCACG |
|  |  | 29) | PDE2_R_WT2 | TCGTCAGTCAATATTTCGGTCCATC |
| qRT-PCR | *actA* | 30) | ARTPCRFW | GCTCCAAGAGCTGTTTTCCCTTC |
|  |  | 31) | ARTPCRRV | GCTTGGATTGCGCTTCATCTC |
|  | *tub* | 32) | TBRTPCRFW | ACATGCTCTGCCATTTTCCG |
|  |  | 33) | TBRTPCRRV | TTGTTAGGGATCCACTCAACGAAG |
|  | *elongation factor* | 34) | EFRTPCRFW | ACTATGTTACCGTCATTGATGCCC |
|  |  | 35) | EFRTPCRRV | ACCAGTACCAGCGGCAATG |
